# Is There Any Mosaicism in *REarranged During Transfection* Variant in Hirschsprung Disease’s Patients?

**DOI:** 10.3389/fped.2022.842820

**Published:** 2022-03-10

**Authors:** Kristy Iskandar, Susan Simanjaya, Taufik Indrawan, Alvin Santoso Kalim, Marcellus, Didik Setyo Heriyanto, Gunadi

**Affiliations:** ^1^Department of Child Health/Genetics Working Group, Faculty of Medicine, Public Health and Nursing, Universitas Gadjah Mada/UGM Academic Hospital, Yogyakarta, Indonesia; ^2^Pediatric Surgery Division, Department of Surgery/Genetics Working Group, Faculty of Medicine, Public Health and Nursing, Universitas Gadjah Mada/Dr. Sardjito Hospital, Yogyakarta, Indonesia; ^3^Department of Anatomical Pathology/Genetics Working Group, Faculty of Medicine, Public Health and Nursing, Universitas Gadjah Mada/Dr. Sardjito Hospital, Yogyakarta, Indonesia

**Keywords:** Hirschsprung disease, *RET* rs2435357 variant, pathogenesis, somatic mosaicism, specific tissue expression

## Abstract

**Background:**

Hirschsprung disease (HSCR) is a heterogeneous genetic disease characterized by the absence of ganglion cells in the intestinal tract. The *REarranged during Transfection (RET)* is the most responsible gene for its pathogenesis. RET’s somatic mosaicisms have been reported for HSCR; however, they are still under-recognized. Therefore, we determined the frequency of somatic mutation of *RET* rs2435357 in HSCR patients at our institution.

**Methods:**

We performed *RET* rs2435357 genotyping from 73 HSCR formalin-fixed and paraffin-embedded (FFPE) rectal and 60 non-HSCR controls using the PCR-RFLP method. Subsequently, we compared those frequencies of genotypes for *RET* rs2435357 with our previous genotyping data from 93 HSCR blood specimens.

**Results:**

The frequencies of genotypes for *RET* rs2435357 in HSCR paraffin-embedded rectal were CC 0, CT 11 (15%), and TT 62 (85%), whereas their frequencies in HSCR blood samples were CC 4 (4.3%), CT 22 (23.7%), and TT 67 (72%). Those frequencies differences almost reached a significant level (*p* = 0.06). Moreover, the frequency of *RET* rs2435357 risk allele (T) was significantly higher in HSCR patients (135/146, 92.5%) than controls (46/120, 38.3%) (*p* = 3.4 × 10^–22^), with an odds ratio of 19.74 (95% confidence interval = 9.65–40.41).

**Conclusion:**

Our study suggests somatic mosaicism in HSCR patients. These findings further imply the complexity of the pathogenesis of HSCR. Moreover, our study confirms the *RET* rs2435357 as a significant genetic risk factor for HSCR patients.

## Introduction

Hirschsprung disease (HSCR) is the leading cause of functional intestinal obstruction in neonates, with 15, 28, and 21 cases per 1,00,000 live births in the European, Asian, and African populations (1, 2). It is caused by the failure of migration, proliferation, and differentiation of neural crest cells during enteric nervous system development (1, 2).

At least 24 genes play a role in the pathogenesis of HSCR, with *REarranged during Transfection (RET)* as one of the significant genes (1–3). Given the heterogeneity of the genes mentioned above, most genes only have a small effect on the formation of HSCR, which is not more than 20 percent of all patients (1, 2, 4). On the other hand, polymorphism on the intron 1 enhancer gene, *RET* rs2435357, is found in ∼80% of patients with HSCR (4). This variant is more commonly found with up to 60 percent of patients without a mutation in the coding sequence of *RET* compared to 14% of patients with a mutation on the coding sequence of *RET*, such that it is said to be a significant risk factor for male patients with isolated S-HSCR (4). Our previous studies showed that *RET* rs2435357 variant is a significant risk factor toward the development of the HSCR in Indonesia (5–7).

Several studies suggested the role of somatic mosaicism in HSCR (8, 9). However, the evidence is still limited and controversial (10, 11). Therefore, we aimed to investigate the frequency of somatic mutation of *RET* rs2435357 in HSCR patients at our institution.

## Materials and Methods

### Samples

Our samples were the paraffin blocks of rectal tissue from 73 HSCR patients <18 years old and 60 non-HSCR patients <18 years old at our institution. This study was approved by the Medical and Health Research Ethics Committee, Faculty of Medicine, Public Health and Nursing, Universitas Gadjah Mada/Dr. Sardjito Hospital, Yogyakarta, Indonesia (Ref. KE/FK/0855/EC/2017). The research has been performed following the Declaration of Helsinki.

### DNA Isolation and Genotyping

Genomic DNA was extracted from the formalin-fixed and paraffin-embedded (FFPE) rectal of HSCR patients and non-HSCR patients using the QIAmp DNA Mini Kit (QIAGEN, Hilden, Germany). For comparison, we used our previous genotyping data of *RET* rs2435357 of HSCR patients from blood samples (6). The blood samples and rectal samples were from the same HSCR patients. The HSCR patient samples were from the full-thickness rectal biopsies.

**FIGURE 1:**
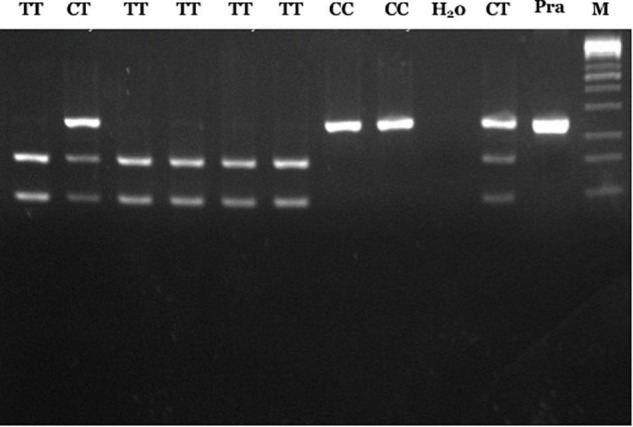
PCR-RFLP results of *RET* rs2435357 variant. Lane 1, 3–6: TT genotype (156 and 90 bp); lane 2, 10: CT genotype (246, 156, and 90 bp), lane 7–8: CC genotype (246 bp), lane 9: H_2_0, lane 11: pra-digested PCR, and lane M: 100 bp DNA marker.

According to our previous study, genotyping of *RET* rs2435357 variant was done using the PCR-RFLP method using forward primer 5′-gagtgcatggggacagtt-3′ and reverse primer 5′-ggaaactgccaattaggttat-3′ (6). The PCR condition was 95°C for 5 min, 35 cycles (95°C for 1 min, 58°C for 1 min and 72°C for 1 min) and using the PCR Swift Maxi thermal cycler (Esco Micro Pte. Ltd., Singapore). After that, the PCR product was digested using restriction enzyme endonuclease *Hin*1II (6, 12). The risk T allele will form a restriction site for the abovementioned enzyme to produce a fragment of 156bp and 90bp, whereas the non-risk C allele does not have a restriction location such that it will only produce one fragment of 246bp. Thus, genotype CC will show one band (246bp), CT with three bands (246bp, 156bp, and 90bp), and TT with two bands (156bp and 90bp) on the 3% agarose gel and visualized using ethidium bromide (Figure 1).

### Statistical Analysis

The association between *RET* rs2435357 and the risk of HSCR was determined using Chi-square or Fisher Exact test with a significance of *p* < 0.05.

## Results

### Comparison of REarranged During Transfection rs2435357 Genotyping in Hirschsprung Disease Patients Between Rectal and Blood Samples

Firstly, we compared our previous genotype of *RET* rs2435357 from the blood samples (6) with the rectal tissue. The frequencies of genotypes for *RET* rs2435357 in HSCR paraffin-embedded rectal tissue were CC 0, CT 11, and TT 62, whereas their frequencies in HSCR blood samples were CC 4, CT 22, and TT 67. Those frequencies differences almost reached a significant level (*p* = 0.06) (Table 1).

**TABLE 1:** Comparison of *RET* rs2435357 genotype in HSCR patients between rectal and blood samples (6).

	Genotype			*p*-value
**HSCR patients**	**CC (n,%)**	**CT (n,%)**	**TT (n,%)**	
Blood sample	4 (4.3)	22 (23.7)	67 (72)	0.06
Rectal tissue	0	11 (15)	62 (85)	

### Association Between RET rs2435357 and Risk of Hirschsprung Disease

Next, we determined the association between *RET* rs2435357 and the risk of HSCR in our population. The frequency of *RET* rs2435357 risk allele (T) was significantly higher in HSCR patients (135/146, 92.5%) than controls (46/120, 38.3%) (*p* = 3.4 × 10^–22^), with an OR of 19.74 (95% CI = 9.65–40.41) (Table 2). Subsequently, we combined the genotype of *RET* rs2435357 from rectal and blood samples6 and associated them with the risk of HSCR. Again, the T allele was significantly associated with the HSCR with the OR of 8.11 (95% CI = 5.53–11.88); *p* = 3.7 × 10^–32^) (Table 3).

**TABLE 2:** Association between *RET* rs2435357 and risk of HSCR in our study.

	Rectal tissue		OR (95% CI); *p*	
Genotype	Cases (n,%)	Controls (n,%)	Dominant (TT + CT *vs.* CC)	Recessive (TT *vs.* CT + CC)
CC	0	6 (10)	17.53 (0.97–317.92); 0.007*	11.27 (4.88–26.01); 1.1 × 10^–9^*
CT	11 (15)	34 (56.7)		
TT	62 (85)	20 (33.3)		
Allele				
C	11 (7.5)	74 (61.7)	19.74 (9.65–40.41); 3.4 × 10^–22^*	
T	135 (92.5)	46 (38.3)		

*Significance p < 0.05; CI, confidence interval; HSCR, Hirschsprung disease; OR, odds ratio.

**TABLE 3:** Association between *RET* rs2435357 and risk of HSCR from all samples.

	Rectal + Blood samples		OR (95% CI); *p*	
Genotype	Cases (n,%)	Controls (n,%)	Dominant (TT + CT *vs.* CC)	Recessive (TT *vs.* CT + CC)
CC	4	32	7.90 (2.73–22.85); 4.9 × 10^–6^*^	2.8 × 10^–25^*^
CT	33	117		
TT	129	47		
Allele				
C	41	209	8.11 (5.53–11.88); 3.7 × 10^–32^*^	
T	291	183		

*Significance p < 0.05; CI, confidence interval; HSCR, Hirschsprung disease; OR, odds ratio.

## Discussion

Our study shows that somatic mosaicism might occur in HSCR patients. The role of somatic mosaicism in HSCR is still controversial. While several studies suggested the somatic mosaicism in HSCR (8, 9, 11), a previous report did not (10). Therefore, our findings provided new evidence of somatic mosaicism in HSCR pathogenesis from a different ethnic group, i.e., Javanese, Indonesia. Interestingly, different findings of somatic mosaicism are noted even among the same population, i.e., Chinese (9–11). While two studies from the Chinese population supported somatic mosaicism (9, 11), one report did not (10). As Indonesia consists of more than 375 ethnic groups (5), further studies with a specific another ethnic group in Indonesia are mandatory to clarify the role of somatic mosaicism in the pathogenesis of HSCR in Indonesia. Another difference in our study from previous reports is that we used PCR-RFLP for genotyping of *RET* rs2435357 in HSCR patients (*vs.* TaqMan method (10) *vs.* Sequencing (8). This method has been shown accurate and more affordable than the TaqMan technique to genotype *RET* rs2435357 in HSCR patients (6).

Most studies of somatic mosaicism focus on cancer (13–15). Somatic mosaicism has been reported in genetic diseases, either Mendelian or complex genetic disorders (8, 9, 11, 14). Somatic mosaicism causes a milder phenotype in Mendelian disorder (14). Whether the somatic mosaicism also results in a milder phenotype in complex genetic disorders such as HSCR is exciting and essential to investigate.

Several pathways have been proposed for the HSCR pathogenesis, including the RET/GFRα1/GDNF, EDNRB/ECE1/EDN3, SOX10/PHOX2B, and SEMA3 (1, 2). However, variants in those pathways attribute to only 20% of all HSCR cases, implying that other mechanisms are supposed to be involved in the pathogenesis of HSCR (16), including somatic mosaicism (9, 11). Although Jiang et al. (9) showed that the somatic mutation of *RET* has a role in the pathogenesis of HSCR, however, another report did not fully agree with those findings (17). They suggested that to determine the somatic mosaicism in HSCR patients; the study should compare the variants between different tissues representing different germ layers, such as blood and colon tissue (17). Our study compared the frequency of *RET* rs2435357 variant in HSCR patients from blood and rectal tissue.

Moreover, determining the somatic mosaicism in HSCR is essential to explain the occurrence of HSCR in the absence of inherited or *de novo* variants during the counseling to the families (18). While the germ-line somatic mutation can be transmitted, the tissue-specific somatic mutation is not (18). A current study failed to identify somatic mosaicism in a small number of HSCR patients. They suggested that it is challenging to find the somatic variants involved in HSCR because these mutations will lead to a selective disadvantage for the affected cell (18).

This study focused on the *RET* rs2435357 variant since this variant has been a vital genetic risk factor for HSCR across populations, including Indonesia (4–7, 19–21). Our current study also supports the *RET* rs2435357 as a significant risk factor for HSCR (Tables 2, 3). *RET* rs2435357 reduces the binding of the critical transcription factor (TF) SOX10 necessary for ganglionosis during the enteric nervous system development (4). These mechanisms are in harmonizing with two other enhancers in *RET*: one binding TF GATA2 and the other binding TF RARB (22). In addition, recent meta-analysis studies showed that besides *RET* rs2435357, other variants in *RET* also increased HSCR risk, including rs1800858, rs1800861, and rs10900297 (23, 24). Further study is necessary to investigate the somatic mosaicism of those three *RET* variants to confirm our findings.

Notably, we extracted the DNA from the FFPE rectal samples. It might result in non-reproducible sequence artifacts (10). In addition, we genotyped the *RET* rs2435357 variant from the rectal samples only. Further study is necessary to use the fresh tissue and compare the somatic mosaicism status between aganglionic, ganglionic, and transitional colon samples.

## Conclusion

Our study suggests somatic mosaicism in HSCR patients. These findings further imply the complexity of the pathogenesis of HSCR. Moreover, our study confirms the *RET* rs2435357 as a significant genetic risk factor for HSCR patients.

## Data Availability Statement

The original contributions presented in the study are included in the article/supplementary material, further inquiries can be directed to the corresponding author.

## Ethics Statement

The studies involving human participants were reviewed and approved by the Medical and Health Research Ethics Committee, Faculty of Medicine, Public Health and Nursing, Universitas Gadjah Mada/Dr. Sardjito Hospital. Written informed consent to participate in this study was provided by the participants’ legal guardian/next of kin.

## Author Contributions

KI and Gunadi conceived the study. KI, Gunadi, SS, and Marcellus drafted the manuscript. SS, TI, and Marcellus collected the data. Gunadi analyzed the data. KI, TI, DH, and Gunadi facilitated all project-related tasks. All authors read and approved the final manuscript.

## Conflict of Interest

The authors declare that the research was conducted in the absence of any commercial or financial relationships that could be construed as a potential conflict of interest.

## Publisher’s Note

All claims expressed in this article are solely those of the authors and do not necessarily represent those of their affiliated organizations, or those of the publisher, the editors and the reviewers. Any product that may be evaluated in this article, or claim that may be made by its manufacturer, is not guaranteed or endorsed by the publisher.
